# Astragaloside IV Reduces Cerebral Ischemia/Reperfusion-Induced Blood-Brain Barrier Permeability in Rats by Inhibiting ER Stress-Mediated Apoptosis

**DOI:** 10.1155/2020/9087873

**Published:** 2020-10-26

**Authors:** Bonan Hou, Rui Liu, You Wu, Shuiqing Huang

**Affiliations:** ^1^Department of Neurology, The Second Affiliated Hospital of Zhejiang Chinese Medical University, Hangzhou, Zhejiang, China; ^2^Department of Chinese Medicine, The Second Affiliated Hospital of Zhejiang Chinese Medical University, Hangzhou, Zhejiang, China; ^3^Science and Technology Innovation Center, Guangzhou University of Chinese Medicine, Guangzhou, Guangdong, China

## Abstract

**Background:**

Previous studies proved that AS-IV could prevent blood-brain barrier (BBB) against an increase in permeability. However, its underlying molecular mechanism has not been enlightened yet. The aim of the study is to reveal the potential protective mechanism of astragaloside IV (AS-IV) on the blood-brain barrier after ischemia-reperfusion.

**Methods:**

In vivo, AS-IV neurological protection was measured by Long's five-point scale and 2,3,5-triphenyltetrazolium chloride staining. AS-IV protection for BBB was observed by Evans blue extravasation technique. Endoplasmic reticulum stress and apoptosis-related protein levels were measured by western blot with AS-IV intervention. In vitro, cell apoptosis was analyzed by western blot and flow cytometry.Endoplasmic reticulum stress-related protein levels were quantified through western blot.

**Results:**

AS-IV treatment could decrease the infarct size in rats' brain and protect the BBB against Evans blue permeating through brain, after ischemia/reperfusion, significantly. Further, ischemia/reperfusion or oxygen‐glucose deprivation/reperfusion was found to have an increase in endothelial cell apoptosis proteins, such as Bax, Bcl-2, and caspase-3, and endoplasmic reticulum stress-associated proteins, such as phosphorylated PERK and eIF2*α*, Bip, and CHOP, which were attenuated by AS-IV treatment.

**Conclusions:**

AS-IV can effectively protect the blood-brain barrier and reduce the area of cerebral infarction via inhibiting endoplasmic reticulum stress-mediated apoptosis in endothelial cells.

## 1. Introduction

Destruction of the blood-brain barrier (BBB) is a common pathological feature of many nervous diseases [1, 2]. Under physiological conditions, it preserves proper homeostasis of the brain by highly specialized selection [3]. However, BBB dysfunction or structural disruption after cerebral ischemia/reperfusion (I/R) may lead to irreversible neuronal damage and brain dysfunction [4]. Thrombolysis after ischemic stroke or I/R injury leads to the destruction of the BBB, resulting in vasogenic cerebral edema, further increasing the permeability of the BBB, and allowing peripheral immune cells, inflammatory mediators, and toxic molecules to infiltrate ischemia, and the ultimate result of that is the death of ischemic neurons [5, 6].

In fact, the BBB is quite dynamic and has a wide permeability, controlled by intra- and intercellular signaling events among the cells which include ECs, astrocytes, and neurons, in the BBB, and other cells that are in contact with the BBB [7]. ECs play an important role in the dynamic permeability range of the BBB. Therefore, the permeability of the BBB is determined not only by the integrity of interendothelial tight junctions but also by the physiological state of ECs themselves. Accordingly, reducing EC damage and maintaining normal EC function may be a promising therapeutic strategy to alleviate brain I/R injury.

Astragaloside IV (AS-IV), a 3-O-beta-D-xylopyranosyl6-O-beta-D-glucopyranosyl-cycloastragenol, is one of the major active components extracted from *Astragalus membranaceus*. Tremendous studies have shown that AS-IV may facilitate the alleviation of central nervous system diseases, such as multiple sclerosis, brain trauma, and cerebral ischemia or I/R through antioxidant, anti-inflammation, antiapoptosis, or prevent the BBB permeability breakdown [8–10]. Also, some research studies showed that AS-IV can reduce the damage of I/R to the brain, and its mechanism is related to the protection of BBB [11, 12]. However, the exact molecular mechanism of AS-IV in protecting the blood-brain barrier has not yet been established.

In the current study, the preprotection effects of AS-IV on BBB after cerebral I/R in vivo and in vitro were investigated. Our results indicate that AS-IV can attenuate brain I/R-induced increase in blood-brain barrier permeability by inhibiting endoplasmic reticulum stress- (ER stress-) mediated apoptosis of ECs, thereby effectively protecting the blood-brain barrier and reducing the area of cerebral infarction.

## 2. Materials and Methods

### 2.1. Animals and Treatments

Specific pathogen-free male Sprague Dawley (SD) rats (above 15 g), 6 weeks old, were obtained from the Experimental Animal Center of Guangzhou University of Traditional Chinese Medicine (Guangzhou, China). The study was according to the guidelines from the Care and Use of Laboratory Animals approved by the Experimental Animal Ethical Committee of Guangzhou University of Traditional Chinese Medicine (Guangzhou, China).

**Figure 1:**
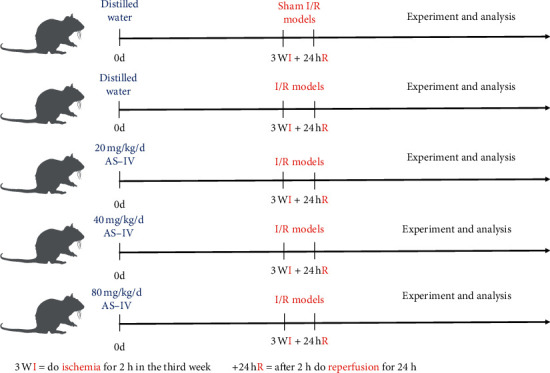
Experimental groups and treatment schedules. AS-IV: astragaloside IV; I/R: ischemia/reperfusion.

The rats were randomly allocated to one of the five groups: sham, model, and three AS-IV groups including AS-20, AS-40, and AS-80. Three AS-IV groups were intragastrically administered with AS-IV (20, 40, and 80 mg/kg/d; purchased from Chengdu Herbpurify Co., Ltd., with the purity of 98.9%), and the sham group and model group were intragastrically administered with distilled water before being established, for 3 weeks (Figure 1).

### 2.2. I/R Models in Rats

Rats were anesthetized with 4% isoflurane until no corneal reflex was observed. Also, 1% isoflurane in a mixture of 30% oxygen and 70% nitrous oxide was used during surgery. The body temperature was controlled at 37°C by an electric blanket. The I/R model was induced by recanalization after occluding the middle cerebral artery via the endovascular technique. Briefly, the rats were fixed in the supine position, and the right side of the neck was fully exposed. A monofilament nylon suture with a slightly enlarged rounded tip was inserted into the stump of the external carotid artery and advanced into the lumen of the internal carotid artery until it reached and occluded the middle cerebral artery for 2 h [13, 14]. The distance from the bifurcation of the common carotid artery to the tip of the inserted suture averaged 18–20 mm. After 2 h after occlusion, the monofilament nylon suture was withdrawn to allow reperfusion for 24 h. The model and three AV-IS rat groups undergo the same protocol. The sham group experienced the same operation except for arterial occlusion.

### 2.3. Neurologic Score of Long's Five-Point Scale

Assessment of animal behavior was performed using Long's five-point scale [15]: the score ranges from 0 to 5 in which the higher the score, the heavier the neurologic deficit. A score of 0 meant no deficit, a score of 4 meant maximal deficit, and a score of 5 meant mortality.

### 2.4. Determination of Infarct Volume

Infarct volumes were measured by using 2, 3, 5-triphenyltetrazolium chloride (TTC) staining (Sigma, St. Louis, Missouri, USA). In brief, at 24 h after reperfusion, the brain was cut coronally into 2 mm thick sections. The coronal sections were subsequently stained with TTC solution at 37°C for 30 minutes.

The infarct size of each 2 mm thick coronal section was calculated by Image-Pro Plus 6.0 (Media Cybernetics, Rockville, USA). Infarct volume calculation is the infarct size of each piece multiplied by the thickness (2 mm) and then added up. The total volume calculation of the brain is the same. Finally, the formula is as follows: infarct volume/the total volume of the brain × 100%.

### 2.5. Evaluation of Vascular Permeability

After being anesthetized, the rats were injected into the tail vein with 2% Evans blue (EB) (1 ml/per rat). 2 hours later, the rats were then transcardially perfused with phosphate-buffered saline until colorless perfusate was observed. The brains were immediately collected, and the hemispheres were separated. Each hemisphere was immersed in formamide for 24 h at 51°C. Formamide was centrifuged for 10 min at 4°C. After that, the absorbance of each sample was measured at 570 nm and 620 nm by a microplate reader (Bio-Rad, USA).

### 2.6. Methods of Euthanasia

In brief, there is a 2-step process. Firstly, rats are first rendered unconscious through exposure to 3% isoflurane and then to 5% isoflurane and subsequently killed via cervical dislocation.

### 2.7. Cell Culture and Treatments

bEnd.3 cells obtained from Guangzhou Jennio Biotech Co., Ltd., were cultured in RPMI-1640 medium with 25 mM HEPES (Wisent Inc., Nanjing, China), 10% fetal bovine serum (FBS, Gibco, USA), and 1% penicillin/streptomycin. All cells were cultured at 37°C in a humidified incubator with 5% CO_2_.

### 2.8. Oxygen-Glucose Deprivation/Reperfusion (OGD/R) and Treatments

The OGD/R model was established as described previously [13, 16]. Before OGD/R, the AS-IV groups' cells were cultured in RPMI-1640 medium with AS-IV (2.5 *μ*M, 5 *μ*M, and 10 *μ*M) for 24 hours. The OGD-treated cells were cultured in glucose-free and serum-free medium, and dishes were placed in a tri-gas incubator (Changsha Huaxing Science and Technology Co, Ltd.) with 5% CO_2_ and 95% N_2_ at 37°C for 4 hours and then recovered in RPMI-1640 medium at 37°C in an incubator humidified with 95% air and 5% CO_2_ for another 4 hours. The control group was cultured in RPMI-1640 medium at 37°C in an incubator humidified with 95% air and 5% CO_2_.

### 2.9. Cell Viability Assay

The viability of cells was measured using the 3-(4,5-dimethylthiazol-2-yl)-2,5-diphenyltetrazolium bromide (MTT) assay. Briefly, cells were received pretreatment with AS-IV and OGD/R and seeded into 96-well plates. Following that, 20 *μ*L MTT (Sigma-Aldrich; Merck Millipore) solution was added to each well and incubated for 4 hours at 37°C. Then the medium was removed, and cells were added with 150 *μ*L DMSO. Finally, the viability of cells was assessed using a microplate reader (Bio-Rad, USA) by measuring the absorbance at 570 nm test wavelengths. The percentage of cell viability was calculated as follows:

(1) $$\begin{array}{c} \text{viability ratio }\left(\%\right)=\frac{\left(A_{570\text{,}\text{ sample}}-A_{570\text{,}\text{ blank}}\right)}{\left(A_{570\text{,}\text{ control}}-A_{570\text{,}\text{ blank}}\right)}\times 100. \end{array}$$

Apoptosis was analysed by Annexin V-FITC/PI double staining [17, 18]. In brief, cells were centrifuged at 2000 rpm for 5 min, washed with cold PBS twice to remove the medium, and suspended with 400 *μ*L 1 × binding buffer. Then, cells were added with 5 *μ*L Annexin V-FITC and incubated at 4°C for 15 min in the dark, followed by incubation with 10 *μ*L PI at 4°C for 5 min in the dark. At last, cell apoptosis was detected using the flow cytometer (Accuri C6, Becton Dickinson Company, USA).

### 2.10. Western Blot Analysis

Proteins were transferred to PVDF membranes and blocked with BSA solution. Then the membranes were incubated with primary antibodies against BIP, CHOP, P-PERK, PERK, P-eif2*α*, eif2*α*i (1 : 1000; CST), Bax, Bcl-2, and caspase-3 (1 : 1000; Boster Biological Technology, Ltd., Wuhan, China) overnight at 4°C. After first incubation, the membranes were washed with TBST and incubated with a horseradish peroxide-conjugated secondary antibody (1 : 3000; Abcam) at 37°C for 1 hour. Protein bands were captured using enhanced chemiluminescent immunoblotting (Bio-Rad, Australia), and protein levels were quantified with Quantity One Software (Bio‐Rad, Hercules, CA).

### 2.11. Statistical Analysis

Data are shown as the means ± standard error of at least three independent preparations. Statistical analysis was performed using the one-way ANOVA test with Stata 7.0 statistical software. *P* < 0.05 was considered statistically significant.

## 3. Results

### 3.1. AS-IV Did Not Reduce Long's Five-point Score

**Figure 2:**
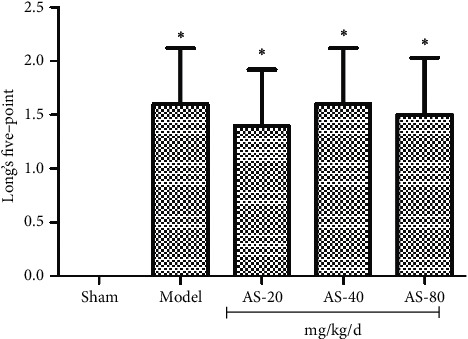
Long's five-point score determined at 24 hours after I/R (*n* = 15). ^*∗*^*P* < 0.01 vs. sham group.

After 24 hours of reperfusion, rats were assessed by Long's five-point score. Except for the sham group, there was no significant difference between the other four groups (Figure 2).

### 3.2. AS-IV Decreased the Infarct Size in Rats' Brain

**Figure 3:**
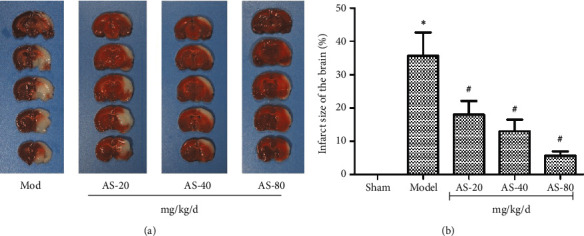
(a) Representative images of 2,3,5-triphenyltetrazolium chloride staining sections after ischemia. (b) Infarct volumes in sham (*n* = 8), model (*n* = 8), 20 mg/kg AS-IV (*n* = 8), 40 mg/kg AS-IV (*n* = 8), and 80 mg/kg AS-IV (*n* = 8) groups. In (b), data are presented as the mean ± standard error of the mean. ^*∗*^*P* < 0.05 and ^*∗∗*^*P* < 0.01 vs. the model group.

AS-IV protected the brain from I/R. The results showed that the infarct volume was 32.40 ± 8.29% in the model group and 3.60 ± 1.34% in the 80 mg/kg AS-IV group. The infarct size of the three drug groups was significantly less than that of the model group, and the difference was statistically significant (ASL: *P* = 0.018; ASM: *P* = 0.006; ASH: *P* = 0.003; Figures 3(a) and 3(b)).

### 3.3. AS-IV Attenuates BBB Disruption in Rats

**Figure 4:**
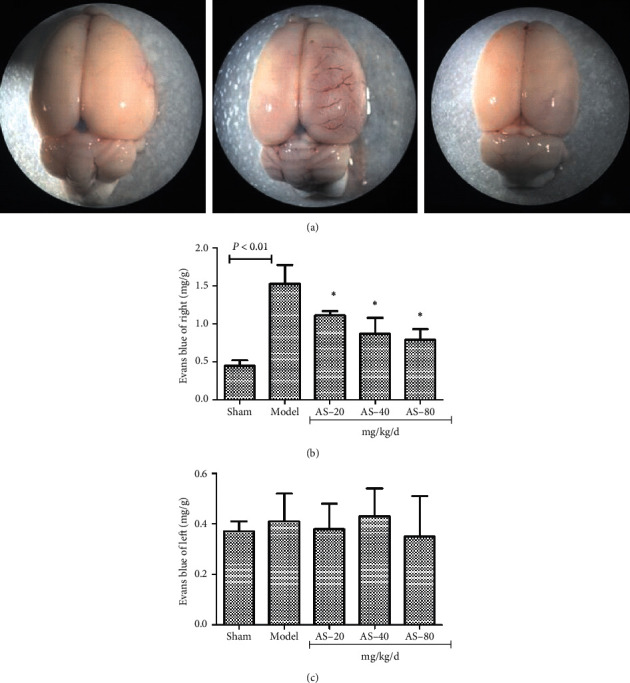
(a) Representative images of EB content of the brain after cerebral I/R. (b) EB content analysis proved that AS-IV attenuates BBB disruption. (c) There is no significance between the five groups. In (b), data are presented as the mean ± standard error of the mean. ^*∗*^*P* < 0.05 vs. the model group.

To determine the therapeutic effect of AS-IV treatment on BBB integrity, we calculated the EB content of the brain (Figure 4(a)). In the right side, cerebral I/R increased the EB content (1.53 ± 0.25 mg/g) in the model group, compared with the sham group. However, comparing the model group and AS-IV groups, 20 mg/kg AS-IV (1.11 ± 0.06 mg/g), 40 mg/kg AS-IV (0.87 ± 0.21 mg/g), or 80 mg/kg AS-IV (0.79 ± 0.14 mg/g) decreased EB extravasation (*P* < 0.05; Figure 4(b)). In the left side of the brain, statistical analysis revealed no significance between the five groups (*P* < 0.05; Figure 4(c)).

### 3.4. AS-IV Ameliorates Cerebral I/R-Induced Cell Apoptosis in Ischemic Hemispheric Tissue

**Figure 5:**
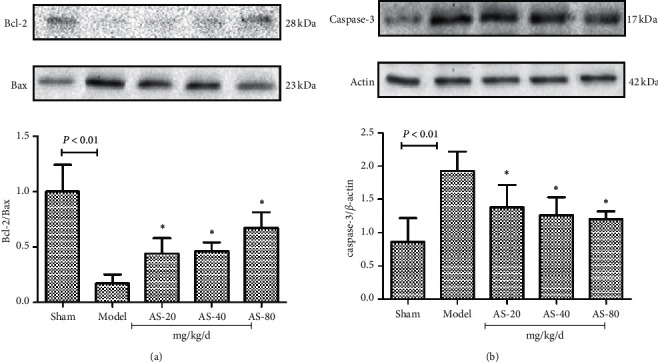
Western blot analysis of apoptosis-related proteins Bcl-2, Bax, and caspase-3 in the brain (a) and their quantification (b) (*n* = 3). ^*∗*^*P* < 0.05 vs. the model group.

In the model group, the protein levels of Bax were increased, while the protein levels of Bcl-2 were decreased. Nevertheless, AS-IV significantly reversed the expression changes of Bax and Bcl-2 (Figure 5(a)). I/R markedly induced the protein levels of caspase-3, which was also inhibited by AS-IV (Figure 5(b)).

### 3.5. AS-IV Inhibited the PERK-eif2*α*-CHOP Signaling Pathway in Ischemic Hemispheric Tissue Induced by Cerebral I/R

**Figure 6:**
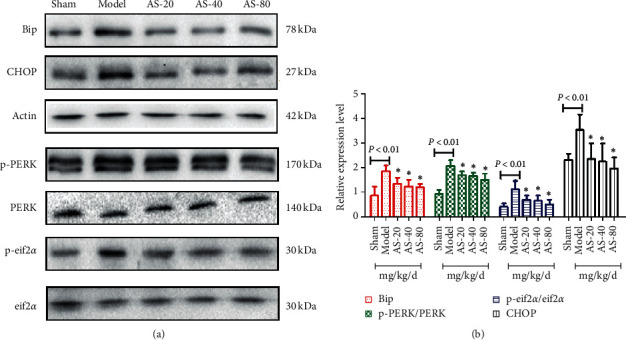
Western blot analysis of ER stress-related proteins p-PERK, p-eif2*α*, CHOP, and Bip in the brain (a) and their quantification (b) (*n* = 3). ^*∗*^*P* < 0.05 vs. the model group.

As shown in Figures 5(a) and 5(b), cerebral I/R injury-induced PERK and eif2*α* phosphorylation increased expression levels of CHOP and Bip significantly. AS-IV can reduce the phosphorylation of PERK and eif2*α* by decreasing the expression of Bip and ultimately inhibit the expression of CHOP. These effects are dose-dependent and are evident at 20 mg·kg^−1^·d^−1^ and peak at 80 mg·kg^−1^·d^−1^ (Figures 6(a) and 6(b)). Our results indicated that attenuation of ER stress levels mediated by AS-IV could reverse cerebral I/R injury.

### 3.6. AS-IV Protect bEnd.3 Cells against OGD/R

**Figure 7:**
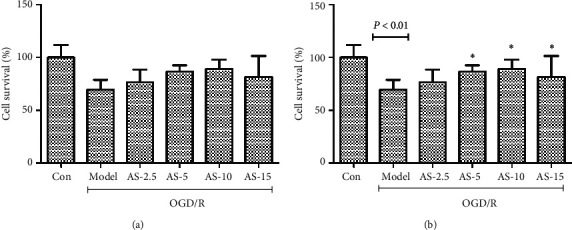
Results of toxicity (a) and protection (b) of AS-IV on bEnd.3 cells.

The effect of AS-IV protected bEnd.3 cells via the MTT assay. The results showed that AS-IV had no toxicity to normal bEnd.3 cells. The effective dose included 2.5 *μ*M, 5 *μ*M, and 10 *μ*M (*P* = 0.002) (Figures 7(a) and 7(b)).

### 3.7. AS-IV Reduced OGD/R‐Induced Cell Apoptosis

**Figure 8:**
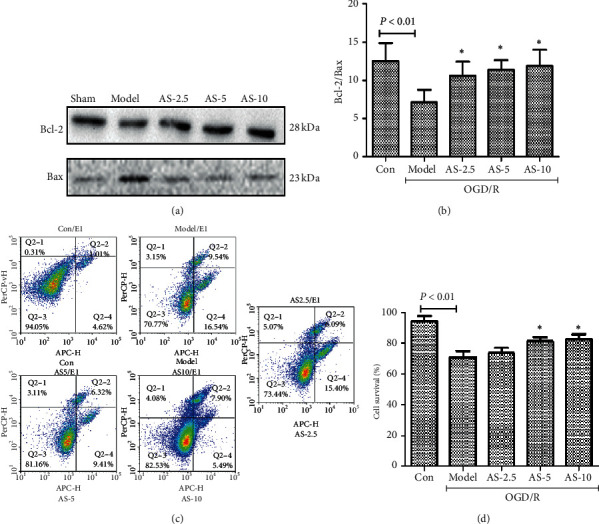
OGD/R induction of bEnd.3 cells apoptosis in vitro. Western blot analysis of apoptosis-related proteins Bcl-2 and Bax after OGD/R (a) and their quantification (b); flow cytometric analysis of apoptosis (c) and (d) (*n* = 5). ^*∗*^*P* < 0.05 vs. the model group.

Western blot analysis showed that after OGD/R, the expression of Bcl-2 was increased and that of Bax was decreased, and this decrease was partly reversed by the administration of AS-IV (Figure 8(a)). These effects are also dose-dependent (Figure 8(b)). Furthermore, flow cytometric analysis revealed a declined apoptosis in OGD/R-induced cells with AS-IV intervention (Figures 8(c) and 8(d)). The results indicated that AS-IV sustained BBB integrity by protecting bEnd.3 cells from apoptosis.

### 3.8. AS-IV Inhibited the PERK-eif2*α*-CHOP Signaling Pathway Induced by OGD/R

**Figure 9:**
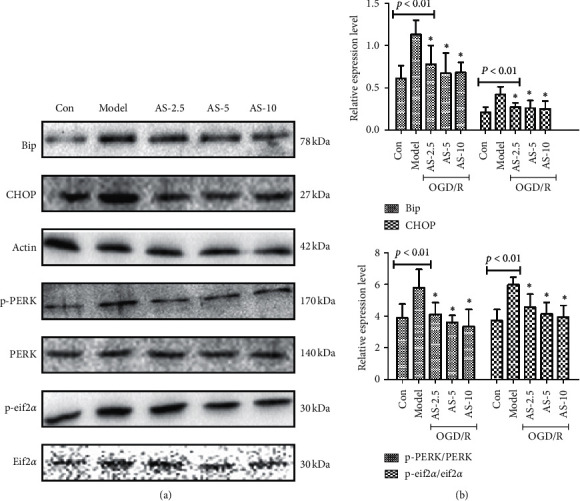
Western blot analysis of ER stress-related proteins p-PERK, p-eif2*α*, CHOP, and Bip in the brain (a) and their quantification (b) (*n* = 5). ^*∗*^*P* < 0.05 vs. the model group.

We validated the role of AS in inhibiting ER stress in the in vitro model (Figure 9). After OGD/R, the expression level of BIP, CHOP, P-PERK, and P-eif2*α* protein was higher (*P* < 0.01). AS-IV significantly relieved ER stress in bEnd.3 cells, with statistically significant differences (*P* < 0.05). Our study suggested that attenuation of ER stress levels mediated by AS-IV could reverse bEnd.3 cells injury that protects BBB integrity.

## 4. Discussion

Dysfunction of BBB is a common pathological feature of many neurological diseases, such as ischemic stroke, hemorrhagic stroke, brain trauma, and neurodegenerative diseases. After thrombolytic therapy for ischemic stroke, the structural and functional disruption of BBB and the resulting cerebral edema are the two most common causes of clinical disability and often indicate poor clinical outcomes [7] because after BBB injury, water, ions, and plasma proteins pass through the BBB interstitial, accompanied by inflammation caused by chemical mediators and cellular infiltration, further aggravating the permeability of BBB. However, current studies about the pathological mechanism of BBB permeability increase after cerebral I/R focus on the tight junction [19].

How about the upstream signaling pathway results in tight junction disruption? The question is still unclear. Chen et al. [20] reported that A*β*_1–42_ induced endothelial cell damage via activation of ER stress in the receptor for advanced glycation end-products-dependent manner and further led to the disruption of the tight junction. Also, mast cell activation contributes to ER stress-mediated endothelial P-selectin expression leading to increased endothelial permeability and impairment of BBB [21], which also indicated ER stress in ECs plays an important role in impairment of BBB. In our current study, we made the following observations: (1) I/R- (OGD/R-) induced brain ECs injury and BBB disruption, accompanied by the activation of ER stress, and (2) ER stress was involved in the disruption of BBB after I/R (OGD/R), and the protective effect of AS-IV involved in inhibiting ER stress after I/R (OGD/R). Taken together, our results suggest that BBB damages reduced by AS-IV inhibiting the activation of ER stress during cerebral I/R (OGD/R).

As a novel subcellular target in the field of ischemic-reperfusion therapy, I/R (OGD/R) can lead to calcium overload and disruption of the endoplasmic reticulum homeostasis, leading to ER stress and prolonged ER stress, eventually leading to apoptosis [22]. Inhibition of ER stress can improve calcium ion overload and oxidative stress to reduce I/R injury [23]. In our present study, we find that I/R (OGD/R) mainly activated the PERK-elF2*α*-CHOP signaling pathway, and AS-IV inhibited ER stress to protect BBB from decreasing phosphorylation level of PERK, in vitro and in vivo. The mechanisms responsible for AS-IV promoting BBB integrity might be inhibition of ER stress by suppressing the upregulation and translocation of NF-kB and reducing the expression and secretion of MMP-9 [24].

AS-IV is the main active ingredient of Radix Astragali. Some researchers suggested that AS-IV protects ECs of podocyte and renal tubular epithelial cells from ER stress-induced apoptosis through downregulating the expression of p-PERK, ATF4, and CHOP [25, 26]. The protective mechanism of AS-IV was mediated at least in part by sarcoendoplasmic reticulum Ca^2+^ ATPase 2b-dependent ER stress attenuation and AMP-activated protein kinase *α*-promoted autophagy induction [27]. Nan et al. studied that ER stress could be a potential therapeutic target to mitigate BBB disruption after I/R (OGD/R). In our study, AS-IV appears to reduce I/R (OGD/R) injury by inhibiting ER stress and thus might be a promising therapeutic agent to protect against BBB damage after acute ischemic stroke or thrombolytic therapy.

It is noteworthy that there is no significant difference in Long's five-point score between the other four groups except the sham group. There were presumable causes that the evaluation interval is too short. We analysed at the first time after reperfusion, which left not enough time to recover. In fact, it will take some days for rats to be neurological rehabilitation [28]. Furthermore, the size of cerebral infarction does not match the degree of neurological deficit clinically. So the different sizes of cerebral infarction could result in the same Long's five-point score.

AS-IV could reduce the expression of BIP, CHOP, P-PERK, and P-eif2*α*, which was caused by I/R (OGD/R). So AS-IV could inhibit the ER stress through inhibiting the PERK pathway of the endoplasmic reticulum and decreasing ECs apoptosis in vitro and in vivo, which protects BBB from increased permeability caused by I/R (OGD/R). However, there are still some problems to be further studied. Such as whether AS-IV significantly improves the neurological deficit of rats or not? We found that AS-IV can maintain mitochondrial function and further speculate whether AS-IV can regulate autophagy. In the mechanism of protecting BBB, is there a relationship between endoplasmic reticulum stress and autophagy? Further study on the unclear problems makes the protection mechanism of AS-IV more clear.

## 5. Conclusion

In conclusion, this study indicates that AS-IV protects BBB through inhibiting the PERK/eIF2*α*/CHOP signaling pathway to reduce the apoptosis of ECs. Our finding shows that AS-IV can protect BBB and reduce the area of cerebral infarction via inhibiting endoplasmic reticulum stress.

## Data Availability

The data used to support the findings of this study are available from the corresponding author upon request.

## Abbreviations

BBB: Blood-brain barrier
AS-IV: Astragaloside IV
ECs: Endothelial cells
I/R: Ischemia/reperfusion
CCA: Common carotid artery
ICA: Internal carotid artery
ECA: External carotid artery
TTC: 2, 3, 5-Triphenyltetrazolium chloride
EB: Evans blue
FBS: Fetal bovine serum
OGD/R: Oxygen‐glucose deprivation/reperfusion
MTT: 3-(4, 5-Dimethylthiazol-2-yl)-2, 5-diphenyltetrazolium bromide.
